# Selective brain cooling achieves peripheral organs protection in hemorrhagic shock resuscitation via preserving the integrity of the brain-gut axis

**DOI:** 10.7150/ijms.61191

**Published:** 2021-06-04

**Authors:** Chien-Ming Chao, Chien-Chin Hsu, Chien-Cheng Huang, Chung-Han Wang, Mao-Tsun Lin, Ching-Ping Chang, Hung-Jung Lin, Chung-Ching Chio

**Affiliations:** 1 Department of Intensive Care Medicine, Chi Mei Medical Center, Liouying, Tainan, Taiwan.; 2 Department of Nursing, Min-Hwei College of Health Care Management, Tainan, Taiwan.; 3 Department of Emergency Medicine, Chi Mei Medical Center, Tainan, Taiwan; 4 Department of Senior Services, Southern Taiwan University of Science and Technology, Tainan, Taiwan.; 5 Department of Environmental and Occupational Health, College of Medicine, National Cheng Kung University, Tainan, Taiwan.; 6 Department of Medical Research, Chi Mei Medical Center, Tainan, Taiwan; 7 Department of Medicine, Taipei Medical University, Taipei, Taiwan.; 8 Division of Neurosurgery, Department of Surgery, Chi Mei Medical Center, Tainan, Taiwan.

**Keywords:** hemorrhagic shock, resuscitation, gut barrier, selective brain cooling

## Abstract

**Background:** Although whole-body cooling has been reported to improve the ischemic/reperfusion injury in hemorrhagic shock (HS) resuscitation, it is limited by its adverse reactions following therapeutic hypothermia. HS affects the experimental and clinical bowel disorders via activation of the brain-gut axis. It is unknown whether selective brain cooling achieves beneficial effects in HS resuscitation via preserving the integrity of the brain-gut axis.

**Methods:** Male Sprague-Dawley rats were bled to hypovolemic HS and resuscitated with blood transfusion followed by retrograde jugular vein flush (RJVF) with 4 °C or 36 °C normal saline. The mean arterial blood pressure, cerebral blood flow, and brain and core temperature were measured. The integrity of intestinal tight junction proteins and permeability, blood pro-inflammatory cytokines, and multiple organs damage score were determined.

**Results:** Following blood transfusion resuscitation, HS rats displayed gut barrier disruption, increased blood levels of pro-inflammatory cytokines, and peripheral vital organ injuries. Intrajugular-based infusion cooled the brain robustly with a minimal effect on body temperature. This brain cooling significantly reduced the HS resuscitation-induced gut disruption, systemic inflammation, and peripheral vital organ injuries in rats.

**Conclusion:** Resuscitation with selective brain cooling achieves peripheral vital organs protection in hemorrhagic shock resuscitation via preserving the integrity of the brain-gut axis.

## Introduction

The causes of hemorrhagic shock (HS), a form of hypovolemic shock in which severe blood loss leads to an inadequate oxygen supply, includes trauma, maternal hemorrhage, gastrointestinal hemorrhage, perioperative hemorrhage, and rupture aneurysm 1. Mild whole-body cooling (WHC) has demonstrated a powerful ability to reduce ischemia-reperfusion injury in HS 2-5. However, the adverse reactions following therapeutic hypothermia (28-32 °C) limit its use in treating HS resuscitation 6. In particular, it might exacerbate coagulopathy in traumatized patients with HS 7.

Hemorrhagic shock or ischemia/reperfusion affected the experimental and clinical bowel disease via the brain-gut axis activation 8. Ischemia/reperfusion after HS severely damaged the gut barrier 9. This raises the possibility that selective brain might improve outcomes of HS resuscitation by preserving the normality of gut barrier permeability.

Infusion of ice-cold saline through internal jugular vein achieved retrograde cerebral infusion achieved selective brain cooling (32 °C) without affecting body core temperature and provided protective effect in ischemic stroke 10, heat stroke 11, and traumatic brain injury 12, 13. However, it is unknown whether the ischemic/reperfusion injury during HS resuscitation can be ameliorated by using selective brain cooling.

The central hypothesis of this study is that intrajugular infusion of cold saline initiated after the onset of blood transfusion resuscitation in a rat HS model will confer peripheral vital organs protection that was achieved by whole-body cooling 2. Following HS, damage to the intestine and other peripheral organs of rats was assessed histologically and biochemically. In particular, intestinal epithelial permeability, systemic inflammation, and peripheral organ injuries were evaluated.

## Materials and Methods

### Animal care

We obtained adult male Sprague-Dawley rats (weight 350±10 g) from BioLASCO Taiwan Co., Ltd., and housed them at an ambient temperature of 24±1 °C with a 12-h light-dark cycle. The Institutional Animal Care and Use Committee (IACUC) of Chi Mei Medical Center approved all animal procedures (IACUC no. 103121505). We used the ARRIVE checklist when writing our report.

### A hemorrhagic shock (HS) model

**Figure 1:**
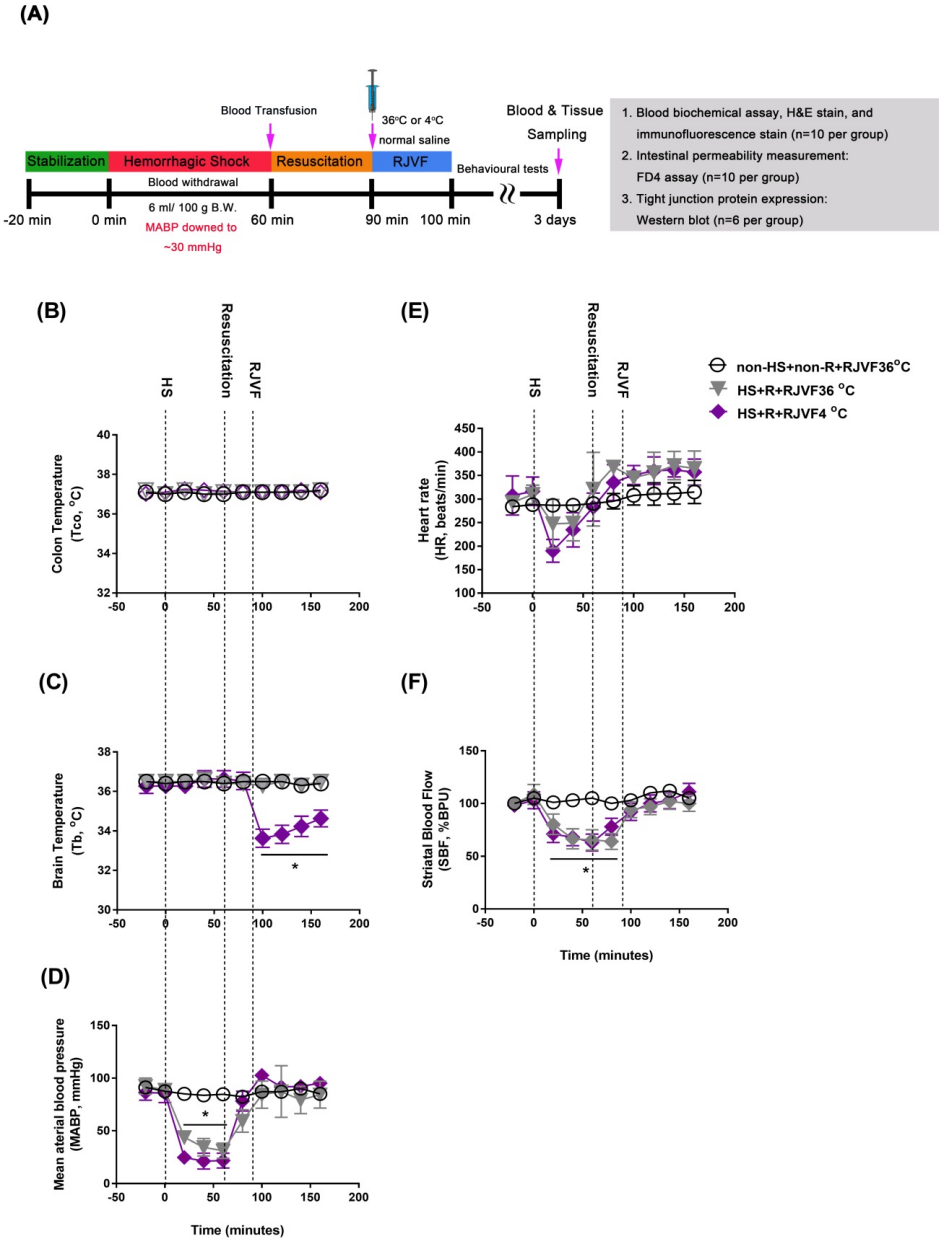
** RJVF 4 °C but not RJVF 36 °C causes selective brain cooling in HS rats. (A)** Illustrations are showing the overall experimental design for the hemorrhagic shock (HS) rat model. **(B)** to** (F)** illustrates **(B)** colon temperature (Tco), **(C)** brain temperature (Tb), **(D)** mean arterial blood pressure (MABP), **(E)** heart rate (HR), **(F)** striatal blood flow (SBF) for different groups of rats (n=10). *p<0.01, compared to the non-HS+non-R+RJVF 36 °C group. The rats (n=78) were divided into 3 groups (n=26 for each group: 10 rats for blood biochemical assay, HE stain, and immunofluorescence stain; 10 rats for intestinal permeability measurement by FD4 assay; 6 rats for intestinal tight junction protein assay by Western blotting;). The overall survival up to 3 days were approximately 10, 8, and 9 out of 10 for non-HS+non-R+RJVF 36 °C, HS+R+RJVF 36 °C, and HS+R+RJVF 4°C, respectively.

All animals were intraperitoneally injected with sodium pentobarbital (40 mg/kg B. W., Sigma-Aldrich, St. Louis, MO, USA) before surgery. In our present study, rats were subjected to an HS as detailed previously 2. In order to induce HS, we withdrew blood (6 mL/100 g B. W.) via the right femoral artery catheter until the mean arterial blood pressure (MABP) dropped to a stable 25 to 30 mmHg for 60 min. After 60 min of HS, we reinfused the shed blood volumes to restore the MABP to its baseline value during a 30-min resuscitation period, followed by selective brain cooling. Sham operation rats underwent similar surgical procedures except for the blood withdrawn and blood transfusion and maintained their MABP within the normal range. Core body temperature (Tco) was continuously monitored in all animals and was maintained at 36 ± 0.2 °C in rat groups without selective brain cooling using an electric heating pad. Ten minutes after the start of brain cooling (that is, 100 min after beginning the induction of HS), the catheter was removed, both the external jugular vein and femoral artery were ligated, and the skin was closed. Animals were returned to their home cages at ambient temperature (Ta, 24 °C) and appropriately fed and hydrated. No supplemental cardiopulmonary support was used. Rats that survived to day three of post-HS were considered survivors, and their data were used for analysis. A schematic diagram of the experimental design is shown in **Figure 1 A**. All the antibodies and commercial kits used in this study are summarized in **Supplementary Table S1**.

### Selective brain cooling by hypothermic retrograde jugular vein flush (RJVF)

After surgery, a Homeothermic control unit (RightTemp® Temperature Monitor & Homeothermic Warming Control Module; Kent Scientific, Torrington, CT, USA) was applied to keep the rectal temperature at 37 ± 0.5 °C. For delivery of cold saline (4 °C, 1.7 mL/100 g B. W) or control vehicle (36 °C, 1.7 mL/100 g B.W.), cannulas (PE50) were inserted into the right external jugular (EJ) vein (with cranial direction). Within the 10-minutes delivery of cold saline, brain temperature was decreased in the rats of the 4 °C RJVF, but their body core temperature remained unchanged 11. After 10-minutes of delivery of cold saline, the brain temperature was maintained at 32-33 °C for at least 50 minutes. Then, the brain temperatures returned gradually to their original levels (as shown in **Figure 1**).

### Experimental groups

The rats (n= 78) were randomized into the following three groups: (i) sham control rats without HS and resuscitation (R) (non-HS+non-R+RJVF 36 °C; n=26); (ii) rats with HS, R, and RJVF 36 °C (HS+R+RJVF 36 °C; n=26); (iii) rats with HS, R, and RJVF 4 °C (HS+R+RJVF 4°C; n=26). The saline (36 °C or 4 °C) was administered via cannulas in the external jugular vein using a syringe pump (KD Scientific Inc., MA, USA). On day 3 post-surgery, animals were subjected to the last behavioral tests. Then they were euthanized with an overdose of Zoletil (100 mg/kg body weight), and 10% Xylazine and their organ were removed for histological and biochemical examinations. The overall survival up to 3 days were approximately 10, 8, and 9 out of 10 for non-HS+non-R+RJVF 36 °C, HS+R+RJVF 36 °C, and HS+R+RJVF 4°C, respectively. Experimenters were blinded as to group allocation.

### Measurement of striatal cerebral blood flow (CBF) in rats under general anesthesia

Upon placing in a stereotaxic apparatus (Model 902, David Kopf Instruments, CA, USA), we inserted an optical probe (300 µm tip diameter; OxyLite^TM^ and OxyFlo^TM^ system; Oxford Optronix Ltd., UK) into the right striatum using coordinates: AP, +0.7 mm distance after the fontanelle; R, +0.3 mm movement to the right side; DV, 6 mm depth value from the top of the skull for monitoring both striatal temperature and striatal CBF with a laser Doppler flowmetry (Oxford Optronix Ltd., UK)^14^.

### Pathohistological and biochemical studies of other vital organs

We evaluated the degrees of brain, lung, liver, kidney, and heart tissue damage according to the descriptions detailed previously 2. We determined the plasma concentrations of blood urea nitrogen (BUN), creatinine, aspartate aminotransferase (AST), alanine aminotransferase (ALT), alkaline phosphatase (ALP), and lactate dehydrogenase (LDH) to assess the renal and hepatic functions and the serum concentrations of tumor necrosis factor-α (TNF-α), interleukin-1β (IL-1β), interleukin-6 (IL-6), and interleukin-18 (IL-18) to assess the inflammatory status.

### Intestinal damage scores and intestinal permeability determination

The duodenum, jejunum, and terminal ileum were fixed with 10% buffered formaldehyde and embedded in paraffin, sliced, stained with hematoxylin and eosin (H&E), and then evaluated the degree of HS-induced damage 15.

Intestinal permeability was determined by measuring the intestinal clearance of fluorescein-isothiocyanate dextran (FD-4; Sigma-Aldrich) as reported in a previous study 16. Briefly, 5 cm segments of duodenum, jejunum, and terminal were collected, and the mucosa was gently everted from each. The intestinal segment was ligated at one end, and from the other end, a gut sac was prepared by injection of 1.0 mL of Krebs-Henseleit bicarbonate buffer. The filled sac was then incubated in a solution containing 0.5 mg/mL FD4 (average molecular weight: 4000) at 37 °C. The bathing solution was also aerated by gently bubbling with a gas mixture containing 5% CO_2_ and 95% O_2_. The mucosal-to-serosal clearance (C) of FD4 was determined in μL-min^-1^•cm^-2^.

### Determination of ZO-1 and claudin-1 immunofluorescence staining at tight junctions of the intestinal epithelium

Intestinal tissues were fixed in 10% formalin overnight and embedded in paraffin. Ten-micrometer intestinal sections mounted on coated glass slides were deparaffinized, rehydrated, and washed with PBS. Antigen retrieval was performed in 0.01 M citrate buffer (pH 6.0) for 30 min in a microwave oven. Slides were incubated with peroxidase blocking reagent (Dako, Carpinteria, CA, USA) for 30 min to block endogenous peroxidase activity and then stained with antibodies targeting claudin-1 (1:200, #2H10D10, Invitrogen^TM^, Thermo Fisher Scientific Inc., Waltham, MA, USA) and ZO-1 (1:200, #ab96587, Abcam, Cambridge, MA, USA) at 4 °C overnight to detect epithelial tight junction proteins as detailed previously 17. Slides were then thoroughly washed with PBS and incubated with Alexa Fluor 488 goat anti-mouse IgG antibody (1:400, # A11008, Invitrogen^TM^, Thermo Fisher Scientific Inc., Waltham, MA, USA) and Alexa Fluor 568 goat anti-rabbit IgG antibody (1:400, #A11004, Invitrogen^TM^, Thermo Fisher Scientific Inc., Waltham, MA, USA) for 1 h at room temperature. The samples were next counterstained with 4'6-diamidino-2-phenylindole (1:5000, #D1306, Invitrogen^TM^, Thermo Fisher Scientific Inc) for 10 min. After a final wash with PBS, slides were mounted in glycerol gelatine mounting medium (#GG1-15 ML, Sigma-Aldrich, St. Louis, MO, USA) and viewed using an upright fluorescence microscope (Carl Zeiss Microscopy GmbH, Jena, Germany) at excitation/emission wavelengths of 578/603 nm (rhodamine, red) and 490/525 nm (FITC, green). A digital camera linked to a computer running Axioscope version 4 (Carl Zeiss) was used to capture images. A pathologist counted the number of ZO-1/DAPI and claudin-1/DAPI double-labeled cells in 20 frames from five sections (x 400 magnification). All cell counting was performed in a blinded manner to avoid bias.

### Western Blot Analysis of Intestinal Tight Junction Proteins Expression

The concentration of the total protein lysates by homogenizing duodenum tissues (n=6 in each group) in RIPA (radioimmunoprecipitation assay) lysis buffer with protease and phosphatase inhibitor (Thermo Fischer Scientific Inc., Waltham, MA, USA) were determined with a protein assay kit (Bio-Rad, Hercules, CA, USA). Twenty micrograms of each protein sample were analyzed by 6% or 12% sodium dodecyl sulfate-polyacrylamide (SDS-PAGE) gels and then transferred to polyvinylidene fluoride (PCDF) membranes. Subsequently, PVDF membranes were incubated with a specific primary antibody to detect rat claudin-1 or ZO-1 protein (#ab15098, #ab96587, Abcam, Cambridge, MA, USA) overnight at 4 °C. β-actin (#sc47778, Santa Cruz, CA, USA) was probed as a protein loading control. For secondary antibody, anti-rabbit IgG or anti-mouse IgG conjugated to horseradish peroxidase (HRP) (Cell Signaling Technology, Danvers, MA, USA). Band densities were determined by scanning densitometry (GS-800, BIO-RAD, Hercules, CA, USA). Finally, the signals were detected using enzyme-linked enhanced chemiluminescence (ECL) reagent (Thermo Fischer Scientific Inc., #34080, Waltham, MA, USA). The density of Western blot bands was quantified using an image analysis system (Image Pro-Plus; Media Cybernetics, USA). Claudin-1 and ZO-1 protein levels were determined after normalizing with β-actin.

### Statistics

Data are presented as the mean±S.D. Repeated measures analysis of variance was used to compare serial biochemical data and vital signs. For physiological, behavioral, and biochemical parameters, the data were analyzed by two-way repeated factor analysis of variance. When the analysis of variance showed significant variance, the Tukey-Kramer post hoc test was used. Parameters such as histological scores and the immunefluorescence staining data with non-normal distribution were analyzed by the Kruskal-Wallis test with Dunn's post-hoc test. We used GraphPad Prism (version 7.01 for Windows; GraphPad Software, San Diego, CA, USA) to analyze the data and set the statistically significant level at P < 0.05.

## Results

### RJVF 4 °C but not RJVF 36 °C causes selective brain cooling in HS rats

As shown in **Figure 1**, before HS or sham operation, all three groups of rats had normal environment and physiological values, including colon temperature (Tco, **Figure 1B**), brain temperature in the striatum (Tb, **Figure 1C**), mean arterial blood pressure (MABP, **Figure 1D**), heart rate (HR, **Figure 1E**), and striatal blood flow (SBF, **Figure 1F**). However, 60 min after HS intervention, compared to the non-HS+non-R+RJVF 36 °C group rats, the two groups of HS rats displayed hypotension, bradycardia, and severe cerebral ischemia (**Figure 1**). In particular, compared to the non-HS+non-R+RJVF 36 °C group rats, the HS+R+RJVF 36 °C group rats had a significantly lower percentage of SBF (**Figure 1F**). Immediately after 30 min of resuscitation by blood transfusion, all physiological parameters were restored to pre-HS levels. In RJVF-treated group rats, immediately after resuscitation, an RJVF with saline at either 36 °C or 4 °C was performed for 10 min. Ten minutes of RJVF 4 °C, but not RJVF 36 °C, caused selective brain cooling (Tb decreased to ~32 °C- 33 °C) for approximately 50 min (**Figure 1C**).

### Selective brain cooling attenuates HS-induced intestinal damage and intestinal epithelial hyperpermeability

**Figure 2:**
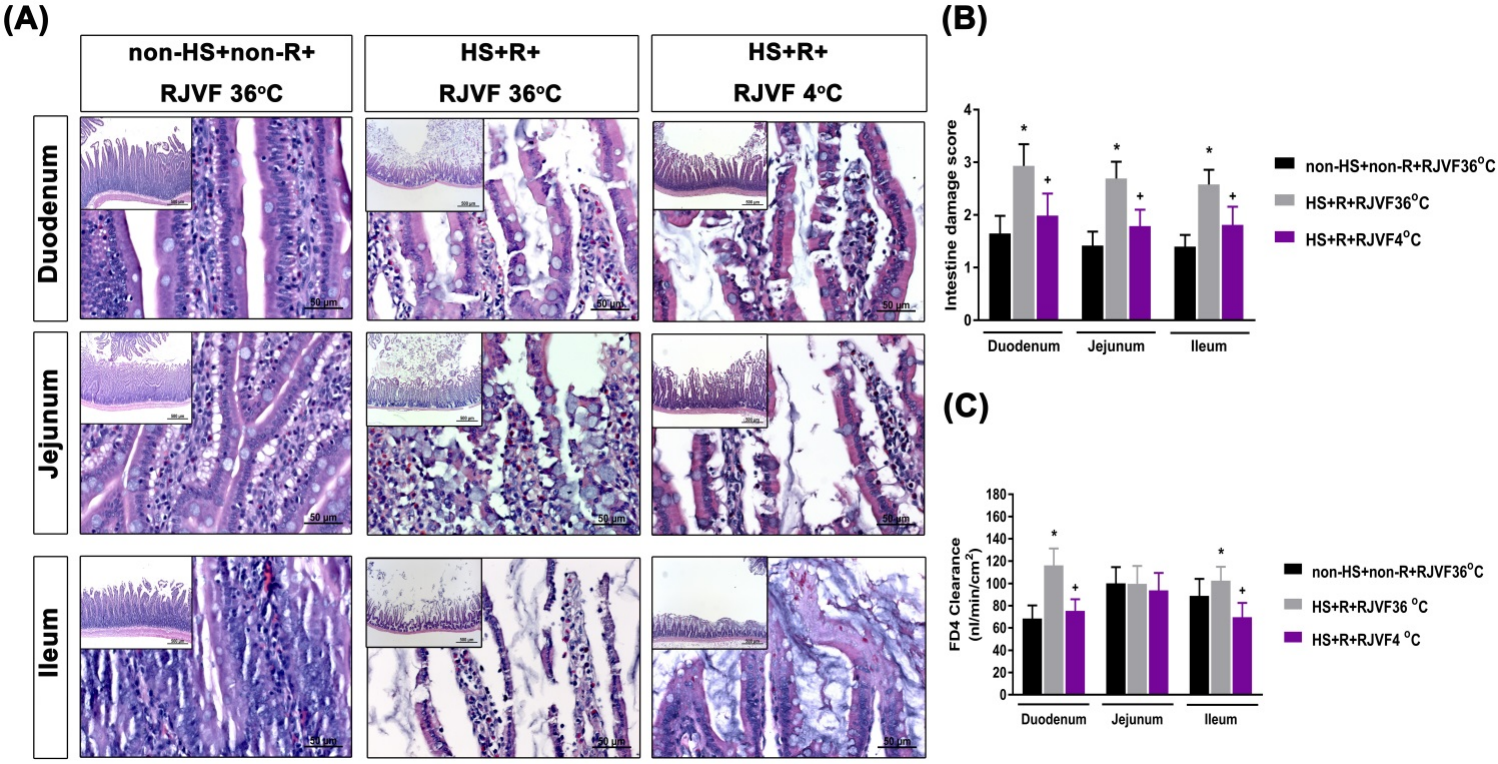
** Selective brain cooling attenuates HS-induced intestinal damage and intestinal epithelial hyperpermeability.** Representative images of the changes to intestinal tissue induced by HS and RJVF as visualized with H&E light microscopy **(A)** and intestinal damage scores **(B)** in each experimental group. **(C)** The intestinal clearance of FD4. Scale bar= 50 µm. The bar graph represents the mean±S.D. of 10 rats for each group. *p<0.05, the HS+R+RJVF 36 °C group vs. the non-HS+non-R+RJVF 36 °C group; ^+^*p*<0.05, the HS+R+RJVF 4 °C vs. the HS+R+RJVF 36 °C group.

**Figure 3:**
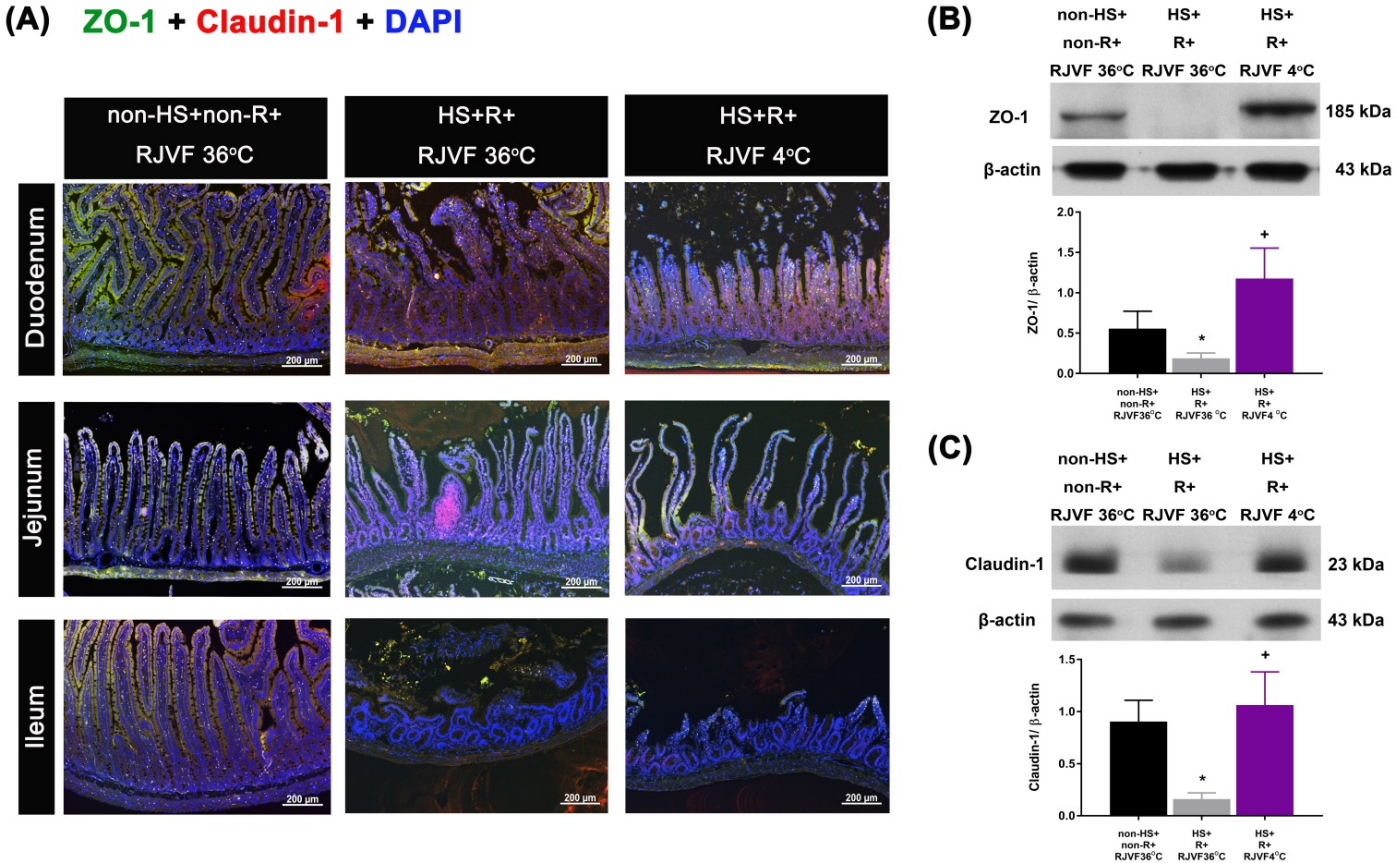
** Tight junction protein expression after hemorrhagic resuscitation.** Representative images of paraffin sections of the intestine labeled for nuclei (blue), **(A)** ZO-1 (green), claudin-1 (red), and merged (yellow). ZO-1 and claudin-1 were detected at the tight junctions of villous enterocytes in each group (n=10). Scale bar= 50 µm. The Western blotting assay revealed the expression levels of **(B)** ZO-1 and **(C)** claudin-1 and a housekeeping protein (β-actin) in the duodenum tissue from each group of rats (n=6). *p<0.05, HS+R+RJVF 36 °C vs. non-HS+non-R+RJVF 36 °C; +*p*<0.05, HS+R+RJVF 4 °C vs. HS+R+RJVF 36 °C.

Compared to the non-HS+non-R+RJVF 36 °C group rats, the HS+R+RJVF 36 °C group rats had significantly higher intestinal damage scores (**Figure 2A-B**). In addition, intestinal epithelial permeability evaluated by both the intestinal clearance of FD4 (**Figure 2C**), the fluorescence intensity of specific intestinal epithelium tight junction proteins (e.g., ZO-1 and claudin-1) (**Figure 3A**), and protein expression levels (**Figure 3B and 3C**) in the HS+R+RJVF 36 °C group rats were also significantly lower than those in non-HS+non-R+RJVF 36 °C group rats. The increased intestinal damage scores, the intestinal epithelial hyperpermeability, and breakdown of tight junction proteins shown in the HS+R+RJVF 36 °C group of rats were all significantly attenuated by selective brain cooling (HS+R+RJVF 4 °C group)(**Figure 2 and 3**).

### Selective brain cooling attenuates multiple vital organs damage in HS rats

**Figure 4:**
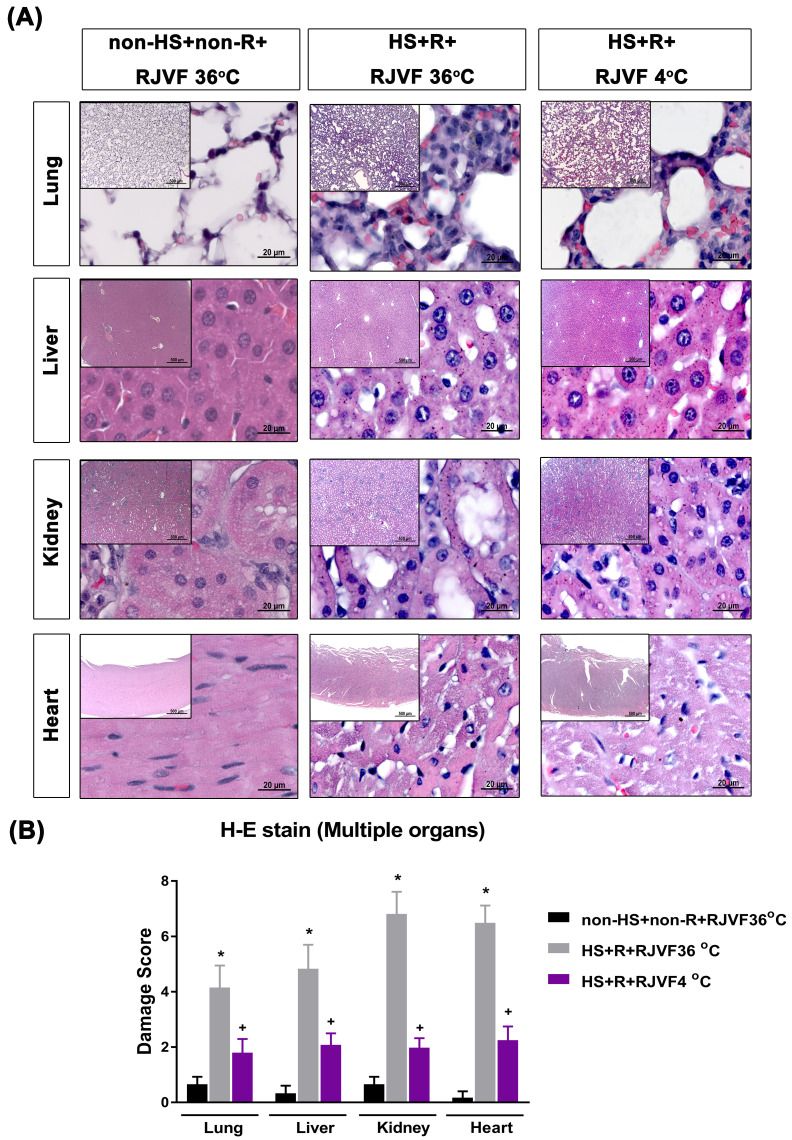
** Multiple organ damage after hemorrhagic resuscitation. (A)** Representative photographs of H & E staining revealed that HS rats showed cell infiltration, pulmonary edema, and alveolar collapse in the lung tissue, disorganized hepatocyte, sinusoidal dilation and necrotic areas in the liver tissues, and altered glomeruli structure (atrophic and dysmorphic), altered tubular structure, altered tubular epithelial cells, vascular dilation/ congestion, increased number of inflammatory cells, intracytoplasmic vacuoles, hemorrhage and inflammatory cell infiltration in the cardiomyocytes. **(B)** The tissue damage scores for different organs. Each bar represents the mean ± S.D. of 10 rats. *p<0.05, the HS+R+ RJVF 36 °C group vs. the non-HS+non-R+RJVF 36 °C group; +*p*<0.05, the HS+R+RJVF 4 °C group vs. the HS+R+RJVF 36 °C group. Scale bar= 500 µm and 20 µm. The images of multiple organs morphology at 50× magnification and 1000× magnification were shown, respectively.

Compared to the non-HS+non-R+RJVF 36 °C group rats, both the HS+R+RJVF 36 °C group rats had significantly higher values of lung, liver, kidney, and heart damage scores (**Figure 4**) and multiple organs damage indicators (e.g., creatinine, BUN, AST, ALT, ALP, and LDH) (**Table 1**). These increased plasma levels of multiple organ damage markers were all significantly reduced by RJVF 4 °C saline but not affected by RJVF 36 °C saline.

### Selective brain cooling attenuates the overexpression of pro-inflammatory cytokines in HS rats

Compared to the non-HS+non-R+RJVF 36 ^o^C group rats, the HS+R+RJVF 36 ^o^C group rats had significantly higher blood levels of pro-inflammatory cytokines (e.g., IL-1β, TNF-α and IL-18) (**Table 1**). The overproduction of pro-inflammatory cytokines was significantly reduced by RJVF 4 °C saline.

## Discussion

Our present result showed that an HS caused systemic inflammation, gut barrier disruption, and peripheral organ damage. Additionally, we found that EJ-based infusion cooled the brain robustly with a minimal effect on body temperatures. This brain cooling significantly reduced systemic inflammation, gut barrier, and peripheral organ damage in HS rats.

Cannulation of the internal jugular vein is a frequently conducted bedside procedure for placing central venous catheters 18. A more recent report showed that internal jugular infusion of cold saline conferred hypothermic and neuroprotective effects in ischemic stroke 10. Compared to the human cerebral venous system, the rat's internal jugular vein is relatively thin for cannulation experiments, and the EJ is the main vessel for cerebral venous drainage, which has a relatively greater diameter for cannulation 11. In this study, we used the external jugular vein for cannulation and found that the cooling and neuroprotective efficacy of the EJ infusion of cold saline was comparable to whole-body cooling by many metrics evaluated 2. Because jugular vein puncture is an easy procedure frequently used in the emergency department, we speculated that hypothermic jugular flush would be an alternative to achieve rapid brain cooling, especially in traumatized patients with an HS 7. However, the EJ is the predominant drainage of the face and head, not the brain. It is bigger than the internal jugular (IJ) vein in non-human mammals because their brains are smaller relative to the face. There are striking differences, including the direction (normal IJ cannulation is antegrade vs. retrograde for this cooling method, and having to accurately direct a catheter towards the cerebrum to deliver cold crystalloid seems much more difficult than placing a standard central venous catheter).

Whole-body cooling (32-34 °C) or therapeutic hypothermia improves the outcome of HS by reducing the resuscitation fluid volume required to maintain blood pressure 19, the expression of reactive oxygen species as well as microvascular permeability 20. Whole-body cooling may exert its beneficial effects by affecting nearly every metabolic, molecular, and cellular event in cell death, ultimately promoting tissue preservation and repair 21. However, the adverse reactions following a whole-body cooling may limit its use in treating HS injury. Probably, the most striking findings of the present study are that EJ infusion of cold saline induces cooling to only the brain (32-33 °C), without the body core (~37 °C), thus circumventing any adverse systemic-effects which may result from total body cooling. Indeed, our present study demonstrates that selective brain cooling induces cooling to the brain, which reduces intestine and other peripheral organ injuries in rats with HS resuscitation. By using the intestine damage scores, we compare the beneficial effects of therapeutic hypothermia between the systemic hypothermia and selective brain cooling. Mucosal damage was classified as follows: 0 (normal), 1 (surface epithelium damaged), 2 (<50% mucosa damaged), 3 (>50% mucosa damaged), and 4 (entire mucosa damaged). Our previous results showed that whole body cooling (32-34 °C) reduced the intestinal damage scores from 3-4 to 2-3 in the rats with HS. In our present results, brain cooling (32-34 °C) shared with whole body cooling the similar efficiency (decreased the intestine damage score from 3-4 to 2-3). The RJVF 4 °C saline infusion can lead to protection with a shorter cooling duration and lower the temperatures by only 3-4 °C. Thus, selective brain cooling would be an attractive alternation to whole body cooling 22, 23. We speculate there is a direct effect to protect organ damage directly from brain specific hypothermia or it is a byproduct of preserving intestinal epithelial hyperpermeability.

Our present study showed that rats with resuscitation from HS exhibited both defective tight junctions (evidenced by altered expression of the transmembrane proteins ZO-1 and claudin-1) and intestinal epithelium hyperpermeability (or increased FD-4 clearance). Activated transcellular mechanisms increase intestinal permeability to macromolecules such as nutrients, proteins, and bacteria 24. In line with our findings, it has been shown that resuscitation from HS or traumatic brain injury causes disruption of the intestinal epithelial barrier accompanied by decreased expression of tight junction proteins ZO-1 and occludin-1 in the intestine 25, 26. In our present results, selective brain cooling attenuates the loss of intestinal epithelial layer integrity, the increased release of pro-inflammatory cytokines, and multiple organ injuries, perhaps via the brain-gut axis 27, 28.

In fact, the “three-hit model” theory proposed by previous studies 9, 27, 29 is partially confirmed by our present results. An initial HS insult causes splanchnic hypoperfusion; the intestine responds by producing and releasing pro-inflammatory cytokines. Blood transfusion resuscitation causes reperfusion and results in both ischemia/reperfusion injury to the intestine and intestinal epithelial hyperpermeability and augments intestinal inflammatory responses. Translocation of bacterias and endotoxins from intestines to the bloodstream further enhances the immune responses with the release of inflammatory cytokines and other mediators both locally and systemically and results in multiple organ deficiency syndromes 27. The central nervous system (CNS) and peripheral tissues are connected by the peripheral nervous system (PNS), which transmits various signals via neurotransmitters to affect immune reactions.

## Conclusions

Following HS, rats caused visceral hypoperfusion, intestinal ischemia/reperfusion injury, gut barrier disruption, systemic inflammation, and peripheral vital organ injuries. Intrajugular-based infusion cooled the brain robustly with a minimal effect on body core temperatures. This brain cooling led to significantly reduced gut barrier disruption, systemic inflammation, and peripheral vital organ injuries in rats. Compared with whole-body cooling, EJ infusion conferred a similar degree of peripheral vital organ protection following HS in rats requiring blood transfusion via preserving the integrity of the brain-gut axis.

## Supplementary Material

Supplementary table S1.

## Figures and Tables

**Table 1:** Indicators of renal, hepatic, and inflammatory function three days after HS or sham intervention.

	Groups		
Indicators	non-HS+non-R+RJVF 36°C	HS+R+RJVF 36 °C	HS+R+RJVF 4 °C
creatinine (mg/dL)	0.38±0.03	0.79±0.06*	0.42±0.04+
BUN (mg/dL)	15±1	31±5*	15±3+
AST (U/L)	96±6	319±14*	82±6+
ALT (U/L)	57±6	187±19*	71±3+
ALP (U/L)	166±53	686±89*	219±17+
LDH (IU/L)	177.66±22.94	525±99*	288±22+
TNF-α (pg/ml)	10±1	32±3*	10±1+
IL-1β (pg/ml)	16±2	29±2*	11±1+
IL-6 (pg/ml)	4±2	22±6*	4±2+
IL-18 (pg/ml)	20±6	59±6*	36±7+

Values are mean±S.D. (n=10 group). *P<0.05 vs. the non-HS+non-R+RJVF 36 °C group; ^+^*P*<0.05 vs. the HS+R+RJVF 36 °C group. HS, hemorrhagic shock; R, resuscitation; RJVF, retrograde jugular vein flush; BUN, blood urea nitrogen; AST, aspartate aminotransferase; ALT, alanine aminotransferase; ALP, alkaline phosphatase; LDH, lactate dehydrogenase; TNF-α, tumor necrosis factor-alpha; IL-1β, interleukin-1beta; IL-6, interleukin-6; IL-18, interleukin-18.

## Abbreviations

ALP: alkaline phosphatase
ALT: alanine aminotransferase
AST: aspartate aminotransferase
BBB: blood-brain barrier
BUN: blood urea nitrogen
CBF: cerebral blood flow
DAPI: 4,5-diamidino-2-phenylindole
EJ: external jugular
ELISA: enzyme-linked immunosorbent assay
FD-4: fluorescein-isothiocyanate dextran
HR: heart rate
HS: hemorrhagic shock
IACUC: institutional animal care and use committee
IJ: internal jugular
IL-1β: interleukin-1 beta
IL-18: interleukin-18
IL-6: interleukin-6
MABP: mean arterial blood pressure
PVDF: polyvinylidene fluoride
RJVF: retrograde jugular vein flush
SBF: striatal blood flow
SDS-PAGE: sodium dodecyl sulfate-polyacrylamide
Tb: brain temperature
Tco: colon temperature
TNF-α: tumor necrosis factor-alpha
WHC: whole-body cooling
