# Supplementary material for: Selective brain cooling achieves peripheral organs protection in hemorrhagic shock resuscitation via preserving the integrity of the brain-gut axis
Source: Int J Med Sci. 2021 Jun 4;18(13):2920–9. doi: 10.7150/ijms.61191 (PMC8241763; doi:10.7150/ijms.61191)
Supplement: Supplementary file 1 — Supplementary table S1. [file ijmsv18p2920s1.pdf]

Supplementary materials

Selective brain cooling achieves peripheral organs protection in hemorrhagic shock resuscitation via preserving the integrity of the brain-gut axis

Chien-Ming Chao[1,2], Chien-Chin Hsu[3], Chien-Cheng Huang[3,4,5], Chung-Han Wang[6], Mao-Tsun Lin[6], Ching-Ping Chang[6,✉], Hung-Jung Lin[3,7,✉], Chung-Ching Chio[8,✉]

[1]Department of Intensive Care Medicine, Chi Mei Medical Center, Liouying, Tainan, Taiwan

[2]Department of Nursing, Min-Hwei College of Health Care Management, Tainan, Taiwan

[3]Department of Emergency Medicine, Chi Mei Medical Center, Tainan, Taiwan

[4]Department of Senior Services, Southern Taiwan University of Science and Technology, Tainan, Taiwan

[5]Department of Environmental and Occupational Health, College of Medicine, National Cheng Kung University, Tainan, Taiwan

[6]Department of Medical Research, Chi Mei Medical Center, Tainan, Taiwan

[7]Department of Medicine, Taipei Medical University, Taipei, Taiwan

[8]Division of Neurosurgery, Department of Surgery, Chi Mei Medical Center, Tainan, Taiwan

Correspondence to:

Ching-Ping Chang, Ph.D., Department of Medical Research, Chi Mei Medical Center. Address: No. 901, Zhonghua Rd, Yongkang District, Tainan City 710, Taiwan.

Phone: +886-6-2812811 ext 52657; fax: +886-6-2832639; e-mail: jessica.cpchang@gmail.com or a50831@mail.chimei.org.tw

Hung-Jung Lin, M.D., Department of Emergency Medicine, Chi Mei Medical Center. Address: No. 901, Zhonghua Rd, Yongkang District, Tainan City 710, Taiwan. Phone: +886-6-2812811 ext 52000; fax: +886-6-2832639; e-mail: 790001@mail.chimei.org.tw

Chung-Ching Chio, M.D., Division of Neurosurgery, Department of Surgery, Chi Mei Medical Center. Address: No. 901, Zhonghua Rd, Yongkang District, Tainan City 710, Taiwan. Phone: +886-6-2812811 ext 52000; fax: +886-6-2832639; e-mail: chiocc@ms28.hinet.net

Supplemental Table 1. Antibodies and kits used in immunofluorescence staining (IF), Western blotting (WB), and ELISA.

Antibody	**Company**	**Titre**	**Catalogue #**	**Purpose**
Claudin-1	Invitrogen<br>Abcam	1:200<br>1:1000	2H10D10<br>Ab15098	IF,WB
ZO-1	Abcam	1:200~1000	Ab96587	IF, WB
β-actin	Santa Cruz	1:5000~10000	sc-47778	WB
Rat IL-1β ELISA kit	R & D Systems	according to the manufacturer protocol	DY501	ELISA
Rat IL-6 ELISA kit	BD Biosciences	according to the manufacturer protocol	550319	ELISA
Rat TNF-α ELISA kit	BD Biosciences	according to the manufacturer protocol	558535	ELISA
Rat IL-18 ELISA kit	Abcam	according to the manufacturer protocol	ab213909	ELISA
4’6-diamidino-2-phenylindole (DAPI)	Thermo Fisher	1: 5000~25000	62247	IF
Secondary antibody Alexa Fluor 488 (rabbit IgG)	Invitrogen	1:400	A11008	IF
Secondary antibody Alexa Fluor 568 (mouse IgG)	Invitrogen	1:400	A11004	IF
Anti-rabbit IgG, HRP-linked	Cell Signaling	1:2000	7074	WB

antibody				
Anti-mouse IgG, HRP-linked antibody	Cell Signaling	1:2000	7076	WB
